# Uniform yolk-shell iron sulfide–carbon nanospheres for superior sodium–iron sulfide batteries

**DOI:** 10.1038/ncomms9689

**Published:** 2015-10-28

**Authors:** Yun-Xiao Wang, Jianping Yang, Shu-Lei Chou, Hua Kun Liu, Wei-xian Zhang, Dongyuan Zhao, Shi Xue Dou

**Affiliations:** 1 Institute for Superconducting and Electronic Materials, Australian Institute of Innovative Materials, University of Wollongong, Innovation Campus, Squires Way, North Wollongong, New South Wales 2500, Australia; 2 Laboratory of Advanced Materials, Shanghai Key Laboratory of Molecular Catalysis, Department of Chemistry, iChEM, Fudan University, Shanghai 200433, China; 3 State Key Laboratory of Pollution Control and Resources Reuse, College of Environmental Science and Engineering, Tongji University, Shanghai 200092, China

## Abstract

Sodium–metal sulfide battery holds great promise for sustainable and cost-effective applications. Nevertheless, achieving high capacity and cycling stability remains a great challenge. Here, uniform yolk-shell iron sulfide–carbon nanospheres have been synthesized as cathode materials for the emerging sodium sulfide battery to achieve remarkable capacity of ∼545 mA h g^−1^ over 100 cycles at 0.2 C (100 mA g^−1^), delivering ultrahigh energy density of ∼438 Wh kg^−1^. The proven conversion reaction between sodium and iron sulfide results in high capacity but severe volume changes. Nanostructural design, including of nanosized iron sulfide yolks (∼170 nm) with porous carbon shells (∼30 nm) and extra void space (∼20 nm) in between, has been used to achieve excellent cycling performance without sacrificing capacity. This sustainable sodium–iron sulfide battery is a promising candidate for stationary energy storage. Furthermore, this spatially confined sulfuration strategy offers a general method for other yolk-shell metal sulfide–carbon composites.

## Abstract (web summary)

There is intensive research into the development of sodium–metal sulfide batteries. Here, the authors report a yolk-shell-like iron sulfide–carbon nanosphere structure as the cathode material which displays exceptionally high performance.

## Graphical abstract

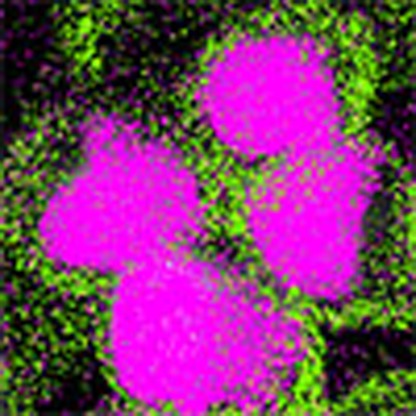

Owing to the increased demand for energy and the need to reduce carbon emissions, energy storage innovation has been a constant global concern over the past decade. Electric vehicles (EVs) and plug-in hybrid EVs are emerging, to reduce our energy dependence on fossil fuels for transportation systems in the future. Lithium-ion batteries (LIBs) have successfully been applied in EV and plug-in hybrid EV trials. Nevertheless, it should be pointed out that concerns about LIBs have arisen both in terms of the high cost and the limitations of lithium resources^1^,^2^. Therefore, research on sodium-based technologies, including Na-ion batteries (NIBs), room-temperature sodium-sulfur batteries (RT-Na/S), and novel Na-O_2_ batteries, has gained momentum due to the overwhelming advantages with regards to the low cost and abundance of sodium resources^3^,^4^,^5^. For NIBs, there is still a long way to go to achieve a sodium-ion full cell system with satisfying energy density and cycling life, as the current research on NIBs has been mainly focused on the search for suitable cathodes^6^,^7^,^8^,^9^,^10^,^11^,^12^,^13^,^14^ and anodes^15^,^16^,^17^,^18^,^19^. For Na-O_2_ batteries, the exploration is only in its initial stage^20^,^21^. Among these sodium-based energy storage systems, the RT-Na/S battery is predicted to deliver high energy density (theoretical value: 760 Wh kg^−1^). Compared with the Li/S battery, however, operation of the Na/S battery at ambient temperature faces a greater critical challenge, because the shuttle effect of sodium polysulfides is much exacerbated, leading to low efficiency and rapid capacity decay on cycling^22^. Even though essential progress has been achieved on the ambient Na/S battery^23^,^24^, the best result can merely reach the energy density of 191 Wh kg^−1^ over 200 cycles. Thus, innovation leading to sodium-based technologies with high energy and power densities is urgently needed.

On the other hand, the emerging sodium–metal sulfide battery has drawn extensive attention owing to its high energy and power densities; the typical cathodes includes iron sulfide (FeS) (ref. 25), FeS_2_ (refs 26, 27), SnS_2_ (ref. 28), MoS_2_ (ref. 29), Ni_3_S_2_ (ref. 30), CuS (ref. 31) and Sb_2_S_3_ (ref. 32). Among all the reported metal sulfides, FeS has risen to prominence, owing to its high theoretical capacity (∼610 mAh g^−1^), high voltage plateau, cost effectiveness, environmental benignity and abundance in nature. The Na/FeS battery, however, shows inferior electrochemical properties due to the detrimental challenges of low conductivity, sluggish kinetics and severe volume changes in the FeS cathode during sodiation/desodiation processes^25^. It is obvious that achieving high capacity is essential and imperative for practical application of the Na/FeS battery. To solve these problems, we designed yolk-shell FeS@carbon nanospheres (FeS@C) as cathode materials. First, the porous carbon shells could enhance conductivity of the active materials, leading to a high reversible capacity. Second, the nanosized FeS cores can offer large electrode/electrolyte contact areas and short diffusion paths for electron and ions, which are favourable to improve the sodium reaction rate, alleviating structural degradation and shortening Na^+^ diffusion paths. More importantly, the suitable void space is able to buffer the large volume variations of FeS during sodiation/desodiation processes, which could maintain the original nanoparticle morphology of the FeS@C and gain prolonged cycling stability^33^. To the best of our knowledge, however, no yolk-shell FeS@C structure has been studied as yet due to the challenges of its synthesis.

Herein we have successfully explored a spatially confined sulfuration strategy for the preparation of uniform FeS@C yolk-shell spheres. Unique FeS@C yolk-shell nanospheres have been configured for the very first time, which consist of single crystalline FeS yolks with an average size of ∼170 nm, specially fabricated void spaces of ∼20 nm and porous carbon shells with a thickness of ∼30 nm. Owing to the multifunctionality of the FeS@C structure, the Na/FeS@C battery is capable of reaching the high capacity of ∼545 mA h g^−1^ over 100 cycles. Furthermore, the remarkable energy density (∼438 Wh kg^−1^) and excellent rate capability (∼452 mA h g^−1^ at 5 C) ensure its great promise for commercial utilization. In addition, the involved Na-storage mechanism has been derived for comprehensive understanding of this promising Na/FeS system.

## Results

### Material synthesis and characterization

**Figure 1:**
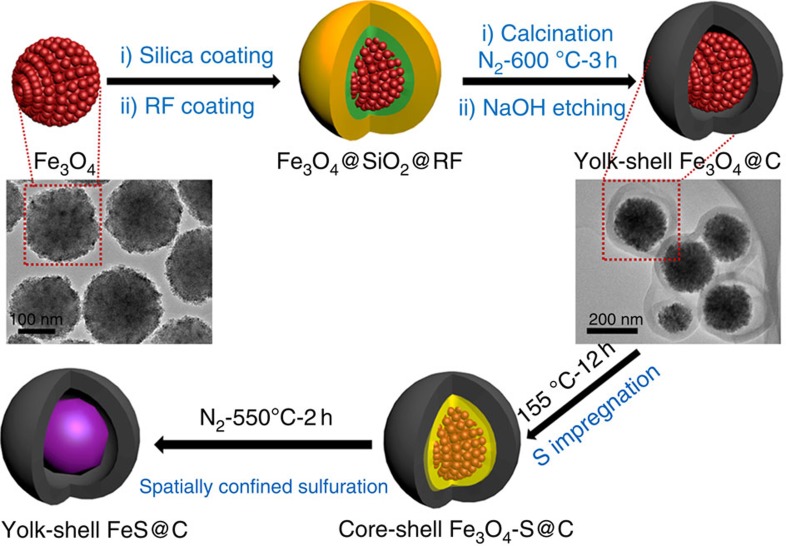
Schematic illustration of the synthesis of yolk-shell FeS@C. TEM images of the Fe_3_O_4_ nanospheres and yolk-shell structured Fe_3_O_4_@C nanospheres have also been inserted in the corresponding positions.

The synthetic procedures for the uniform yolk-shell structured FeS@C nanospheres through a spatially confined sulfuration strategy are illustrated in Fig. 1. First, uniform Fe_3_O_4_ nanoparticles are synthesized, with each of the Fe_3_O_4_ nanoparticles assembled from a number of magnetite nanocrystals, thereby leading to abundant nanopores from the packing. Second, a sacrificial layer of condensed silica coated on the Fe_3_O_4_ nanoparticles via the conventional Stöber method, followed by coating with a polymeric layer of resorcinol formaldehyde (RF) via a sol-gel process. Third, uniform Fe_3_O_4_@C nanospheres with fine yolk-shell structures are obtained through a carbonization process, followed by etching away the sacrificial silica layer with sodium hydroxide (NaOH) solution. Then, there is a crucial step, where sulfur reactant is impregnated into the void spaces of the Fe_3_O_4_@C nanospheres by the melt-diffusion approach and Fe_3_O_4_-S@C mixture nanospheres are constructed. Finally, the uniform yolk-shell FeS@C nanospheres are fabricated via the spatially confined sulfuration strategy based on the sealed solid reaction of Fe_3_O_4_ and the loaded sulfur in the former void space. The elaborate yolk-shell structure of the uniform Fe_3_O_4_@C nanospheres plays a vital role in this strategy. As shown in the transmission electron microscope (TEM) image (Fig. 1), the nanopores (rough size of ∼5 nm) in each Fe_3_O_4_ core with an average diameter of ∼180 nm are beneficial for sufficient sulfur penetration and absorption, to make sure that the formation of FeS particles takes place inside of each carbon shell. Moreover, the void space (∼20 nm) derived from the etching of the silica middle layer also provides sufficient room for sulfur impregnation (Supplementary Fig. 1a,b). On the other hand, the carbon shells (∼30 nm) can confine the reaction zone of the Fe_3_O_4_ and S, and avoid the further growth of the FeS nanoparticles to micron size. Meanwhile, the carbon shells are proven to be porous by nitrogen sorption analysis (Supplementary Fig. 1c,d), which facilitates the penetration of sulfur by the simple melt-diffusion method^34^,^35^. The success of this strategy could be confirmed by the TEM images and the corresponding phase mapping of the Fe_3_O_4_-S@C mixture nanospheres (Supplementary Fig. 2), where the void space is fully filled in by the impregnated solid sulfur, leading to the coexistence of the two phases of Fe_3_O_4_ and S in the carbon shells. For comparison, micron-sized FeS (micro-FeS) particles and core-shell FeS/C nanoparticles were obtained by direct solid reaction of Fe_3_O_4_ nanoparticles and of core-shell Fe_3_O_4_/C nanoparticles with impregnated sulfur, respectively (Supplementary Figs 3 and 4). Assuming the formation of Fe_2_O_3_, SO_2_ and CO_2_ in air atmosphere at 900 °C, the carbon contents of the core-shell FeS/C and the yolk-shell FeS@C are estimated to be 13% and 17%, respectively, according to the thermogravimetric analysis results (Supplementary Fig. 5).

**Figure 2:**
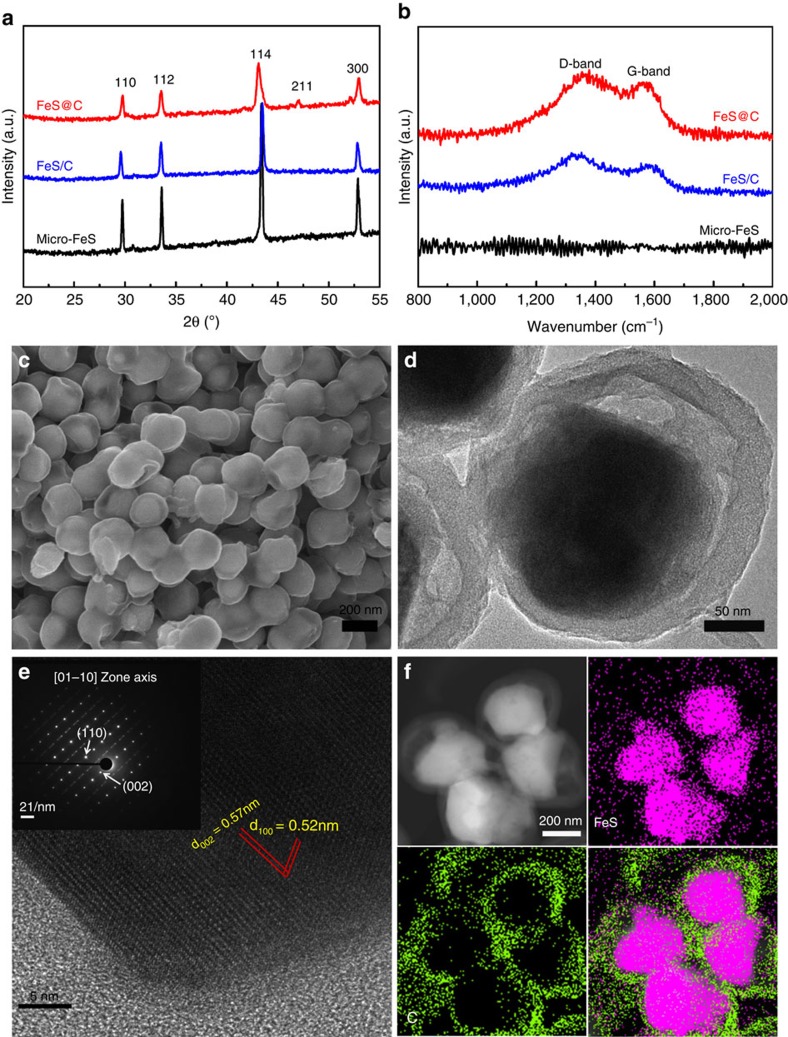
Physical characterization of the three samples. (**a**) XRD patterns and (**b**) Raman spectra of the micro-FeS, core-shell FeS/C nanospheres and yolk-shell FeS@C nanospheres. (**c**) SEM, (**d**) TEM and (**e**) high-resolution TEM images with the corresponding SAED pattern (inset) and (**f**) scanning TEM (STEM) image with phase mapping for the yolk-shell FeS@C nanospheres.

As depicted in Fig. 2a, all X-ray diffraction (XRD) peaks of the micro-FeS, core-shell FeS/C and uniform yolk-shell FeS@C nanospheres can be indexed to troilite crystalline FeS with lattice constants *a*=5.958 Å, *c*=11.740 Å (JCPDF no. 00-037-0477) without impurity, demonstrating that the Fe_3_O_4_ nanoparticles have been successfully reacted with impregnated sulfur to form pure-phase FeS crystals. In contrast, the diffraction peaks of the yolk-shell composites are lower in intensity and more broadened, which indicates the smaller crystalline size of the FeS. No obvious diffraction peak from carbon can be detected, which is ascribed to the amorphous nature of the carbon shells. As further confirmed by the Raman spectra (Fig. 2b), two peaks centred at 1,334 and 1,589 cm^−1^ can be observed for the core-shell FeS/C and the uniform yolk-shell FeS@C nanospheres, corresponding to the characteristic disorder-induced D-band and the graphitic G-band of carbon, respectively. Furthermore, the strong intensity of the D-band indicates that the carbon shells are amorphous. The scanning electron microscope (SEM) image in Fig. 2c shows the high quality of the homogeneous and uniform yolk-shell FeS@C nanospheres. The electron beam could penetrate through the carbon shells, confirming the yolk-shell structure of FeS@C with a particle size of ∼260 nm (outside diameter). It is notable that the FeS@C spheres generally inherit the yolk-shell morphology and diameter of the Fe_3_O_4_@C (Supplementary Fig. 1).The TEM images further reveal that the FeS yolks ∼170 nm in diameter are crystalline single particle and homogeneously encapsulated by the carbon shells (∼30 nm) with void space ∼20 nm in thickness in between (Fig. 2d). Furthermore, the selected area electron diffraction (SAED) pattern in the inset of Fig. 2e displays a set of parallel fringes with *d-*spacing of 0.52 and 0.57 nm, respectively, corresponding to the (100) and (002) planes of single crystalline FeS. The SAED pattern can be indexed as the single-crystal troilite FeS structure with the incident electron beam parallel to the 01–10 orientation as well. Moreover, the phase mapping of the yolk-shell FeS@C nanospheres in Fig. 2f demonstrates that the FeS yolks and the void space results from the interior reaction of Fe_3_O_4_ and S in the core-shell Fe_3_O_4_-S@C mixture (Supplementary Fig. 2). All these results verify that pure-phase FeS encapsulated by porous carbon in a yolk-shell nanosphere has been successfully fabricated via the spatially confined sulfuration strategy.

### Sodium-storage mechanism

**Figure 3:**
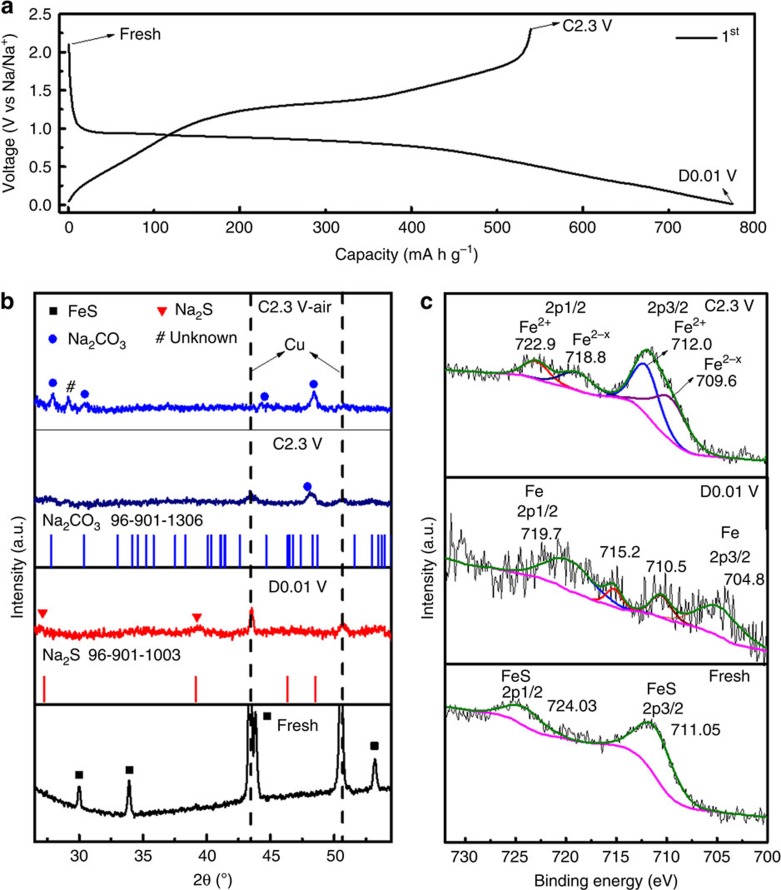
Sodium-storage mechanism of Na/FeS battery. (**a**) Charge/discharge profiles of the micro-FeS electrode for the first cycle at 50 mA g^−1^; (**b**) the corresponding *ex-situ* XRD patterns with the standard XRD patterns of Na_2_S and Na_2_CO_3_ (inset), and (**c**) the respective *ex-situ* Fe 2p XPS spectra of the micro-FeS electrode at the denoted states.

Charge and discharge curves, *ex-situ* XRD and *ex-situ* X-ray photoelectron spectroscopy (XPS) were used to investigate the sodium-storage mechanism of the crystalline FeS particles. The charge and discharge curves of the micro-FeS-based battery at a current density of 50 mA g^−1^ are displayed in Fig. 3a. A distinct plateau occurs at ∼0.85 V for the first discharge process, which is supposed to originate from reactions between FeS and Na, with the formation of Fe, Na_2_S and Na-rich phases corresponding to the quantity of sodiation per FeS (ref. 36). When the cell is discharged to 0.01 V, a further sodiation reaction takes place to form Na_2_S and Fe along with the formation of a solid electrolyte interphase (SEI) film^37^. More evidence is revealed via *ex-situ* XRD at selected typical states: fresh electrode, the first discharge to 0.01 V (D0.01 V) and the first charge to 2.3 V (C2.3 V). The XRD patterns were collected with a scan rate of 0.5 degree per minute from the powders scraped off the electrodes at the selected states. For the XRD pattern at D0.01 V in Fig. 3b, all the conspicuous diffraction peaks of FeS crystals in fresh electrode disappear. Except for two peaks from the copper current collector, only two broad diffraction peaks at 27° and 39.2° are observed, which can be indexed to Na_2_S crystals. The peaks of Fe could not be detected, mostly due to either the ultra-small crystal size or the amorphous nature of resultant Fe nanograins. The *ex-situ* XRD patterns of the Na/micro-FeS battery are similar to those of its Li counterpart^38^, indicating an analogous mechanism based on the conversion reaction. The sodium-storage mechanism is more evident via tracking the variations of the Fe2p peaks during the discharge/charge process. As shown in the *ex-situ* XPS spectra (Fig. 3c), the Fe2p peaks are located at 711.05 and 724.03 eV in fresh electrode, respectively, which originate from FeS (ref. 39). Although the Fe2p signals are weak when the electrode is further discharged to 0.01 V, the peaks for Fe^0^ can be observed at 704.8 and 719.7 eV (ref. 40). The peaks at 710.5 and 715.2 eV should probably be ascribed to the intermediate Na-rich phases. This speculation can be further confirmed via the fringes for metallic Fe in the high-resolution TEM image for the D0.01 V electrode and the high-valence Fe2p peaks in the XPS spectrum after exposing the D0.01 V electrode to air (Supplementary Fig. 6). Therefore, the initial discharge reaction can be expressed as follows:


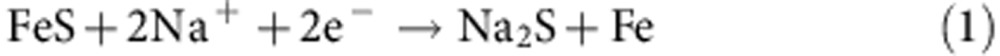


During the following charge process, an obvious platform arises from 1.25 V, which is probably due to the reversible desodiation reaction to Na_2_FeS_2_ (ref. 41). With charging up to 2.3 V, the initial high charge capacity of ∼539 mA h g^−1^ is reached, which corresponds to 1.77 Na ions removed, and the charge product is Na_*x*_FeS (*x*=0.23). In the XRD pattern where the electrode is charged back to 2.3 V, only one diffraction peak appears at 48.3° and no peak from FeS is detected. To index the diffraction peak, the powder was exposed to air overnight (C2.3 V air). It is interesting that the diffraction peak at 48.3° becomes more intense and two more peaks are resolved at 27.8° and 30.4°, which all are well indexed to Na_2_CO_3_. This is further confirmed by the *ex-situ* XPS spectrum of C1s (Supplementary Fig. 7), in which the C1s peak from the O–C=O group becomes more intense for the C2.3 V electrode. The Fe2p peaks of Fe^2+^ are clear in the spectrum for the C2.3 V electrode (Fig. 3c), which results from the desodiation product of Na_2_FeS_2_. The Fe2p peaks at 709.6 and 718.8 eV are mostly due to Fe^2−*x*^ of the Na_*x*_FeS_2_. Therefore, the possible reactions during the reversible desodiation/sodiation processes are listed as follows:


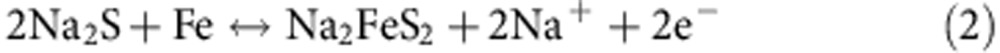



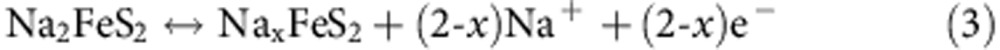


### Electrochemical properties of Na/FeS battery

**Figure 4:**
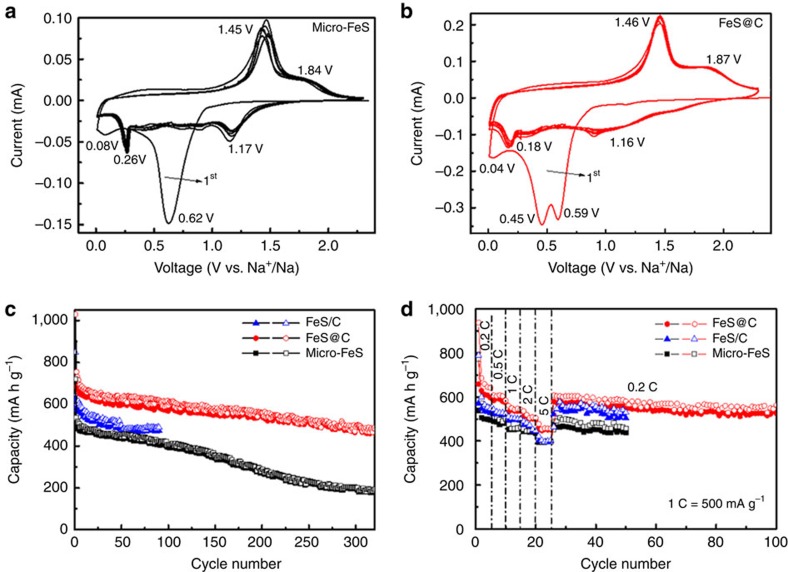
Electrochemical performance of all the samples. Cyclic voltammograms of (**a**) the micro-FeS and (**b**) yolk-shell FeS@C nanospheres at the scan rate of 0.5 mV s^−1^; (**c**) cycling performances at 0.2 C (100 mA g^−1^); and (**d**) rate capability at various current rates of the micro-FeS, core-shell FeS/C nanospheres and yolk-shell FeS@C nanospheres.

The electrochemical performances of both the micro-FeS particles and the yolk-shell FeS@C nanospheres are compared in Fig. 4. Figure 4a,b presents cyclic voltammograms (CVs) of the micro-FeS particles and the yolk-shell FeS@C nanospheres collected at the scan rate of 0.5 mV s^−1^ for ten cylces. During the initial cathodic scan, a large peak at ∼0.6 V appears for the micro-FeS particles and two pronounced peaks at ∼0.45 and 0.59 V occur for the yolk-shell FeS@C nanospheres, which is similar to what is observed in the CV curves of the core-shell FeS/C nanospheres (Supplementary Fig. 8). Even though the peaks are slightly different due to the nanostructure and the introduction of the carbon confinement shells in core-shell FeS/C and yolk-shell FeS@C, the reactions are probably related to the conversion from FeS to Fe and Na_2_S, and the formation of SEI layers on the surface of the FeS and/or C, in agreement with their charge/discharge profiles (Supplementary Fig. 9). For the anodic scan, a sharp peak at ∼1.45 V and a broad one at ∼1.8 V in both samples correspond to the desodiation reaction, where Na_2_FeS_2_ and Na_*x*_FeS species are probably formed, respectively. Similarly, higher anodic potential peaks of Li_2_FeS_2_ and Li_*x*_FeS have been reported to occur at ∼1.9 and 2.3 V in LIBs^42^. For the subsequent cycles, two pairs of cathodic/anodic peaks occur for both electrodes, consistent with the reaction mechanism proposed above. Furthermore, the CV curves of the Na/FeS@C cell show great repetition, indicating its better cycling stability and long lifespan. The cycling performances of the cells based on micro-FeS particles, core-shell FeS/C nanospheres and yolk-shell FeS@C nanospheres are plotted in Fig. 4c. It is manifest that the yolk-shell FeS@C nanospheres outperform the micro-FeS particles and the core-shell FeS/C nanospheres, in terms of both capacity and stability. The micro-FeS delivers a decent electrochemical performance with a stable capacity of ∼400 mA h g^−1^ over 150 cycles. It benefits from the generation of Fe nanograins by the above-discussed conversion reaction, which results in high conductivity of the electrode. Only a low reversible capacity of 195 mA h g^−1^, however, is retained after 300 cycles, which corresponds to a capacity retention of 37.8%. The rapid capacity decay after 150 cycles is due to the possible pulverization of active materials, cracking of the electrode surface and even electrode materials peeling off from Cu current collector during prolonged cycling, which can be affirmed by the corresponding SEM images of micro-FeS electrode after cycling (Supplementary Fig. 10). The core-shell FeS/C nanoparticle electrodes possess higher conductivity and shorter sodium ion diffusion paths due to the synergy between the carbon and nanosize effect, thus resulting in a higher utilization and a larger reaction rate of the active materials, and delivering enhanced capacity (620 and 848 mA h g^−1^ at the first cycle). Nevertheless, its cycling trend is similar to that of the micro-FeS with comparable reversible capacity of 480 mA h g^−1^ after 50 cycles. This indicates that the core-shell structure cannot tolerate the volume expansion of the FeS cores and is easy to rupture. In contrast, when extra voids are created between the FeS nanoparticles and C shells, the yolk-shell FeS@C nanostructure is expected to effectively optimize the electrochemical performance of FeS. The yolk-shell FeS@C nanospheres deliver high initial charge and discharge capacity (722 and 1,029 mA h g^−1^); the reversible capacity higher than the theoretical value is mostly ascribed to the reversible SEI formation and stabilization during the initial cycles. The low Coulombic efficiency (∼70.2%) is ascribed to the irreversible reactions and electrolyte decomposition. A decent capacity of 488 mA h g^−1^ is obtained after 300 cycles, representing a high capacity retention of 67.6%. Along with prolonged cycling, the SEI film layer would become thicker and thicker due to partial reversibility of its formation and stabilization; the yolk-shell structure is prone to be gradually damaged, resulting from the large volume variations of FeS yolk during sodiation/desodiation processes. Therefore, the active materials may lose intact contact from substrate and/or interparticle with increased resistance. All these degenerations lead to the gradual capacity decay of the yolk-shell FeS@C electrode. Their rate capabilities were further investigated at various current densities ranging from 0.2 to 5 C (Fig. 4d). It is noteworthy that the micro-FeS and core-shell nano-FeS show comparable low capacity at the high current rate (2 and 5 C). It indicates that the core-shell structure cannot endure repeated charge/discharge processes, thereby leading to the serious rupture of the structure and subsequent agglomeration of FeS nanoparticles after the initial cycles at the low current rates (0.2, 0.5 and 1 C). The yolk-shell FeS@C shows the most remarkable rate capability. An average reversible capacity of 621 mA h g^−1^ is obtained at 0.2 C. When the current rate increases to 0.5, 1, 2 and 5 C, the recorded capacities reach 584, 537, 505 and 452 mA h g^−1^, respectively. It should be noted that the capacity retention of FeS@C remains as high as 60% when the current rate is increased by 25 times. When the rate is directly reduced to 0.2 C again, the capacity recovers to 584 mA h g^−1^ and remains stable for 100 cycles. This stable trend is in good agreement with its cycling stability, as shown in Fig. 4c. Furthermore, when a fast charge/discharge test (2C=1,000 mA g^−1^) is directly applied, the core-shell FeS/C and yolk-shell FeS@C nanospheres deliver much higher reversible capacity than the microsized FeS over 500 cycles, verifying that the shortened sodium diffusion length and high conductivity is favourable to high-rate reactions (Supplementary Fig. 11). Meanwhile, unlike the significant capacity decay of the micro-FeS and core-shell FeS/C after 150 cycles, it is noticed that the yolk-shell FeS@C nanospheres show preferable cycling stability over prolonged cycles, confirming the superiority of the nanostructured yolk-shell architecture. After the rate capability testing, electrochemical impedance spectroscopy measurements on the cells were conducted (Supplementary Fig. 12). Significantly, the FeS@C after 100 cycles still possesses a lower charge transfer resistance (*R*_ct_) than the micro-FeS after 50 cycles of rate capability testing, implying that there is a favourable SEI film on the FeS@C electrode. The superior electrochemical properties of FeS cathode, therefore, could be gradually achieved via a nanostructuring strategy with respect to nanosized single-crystalline FeS yolks, carbon shells and crucial void space.

## Discussion

**Figure 5:**
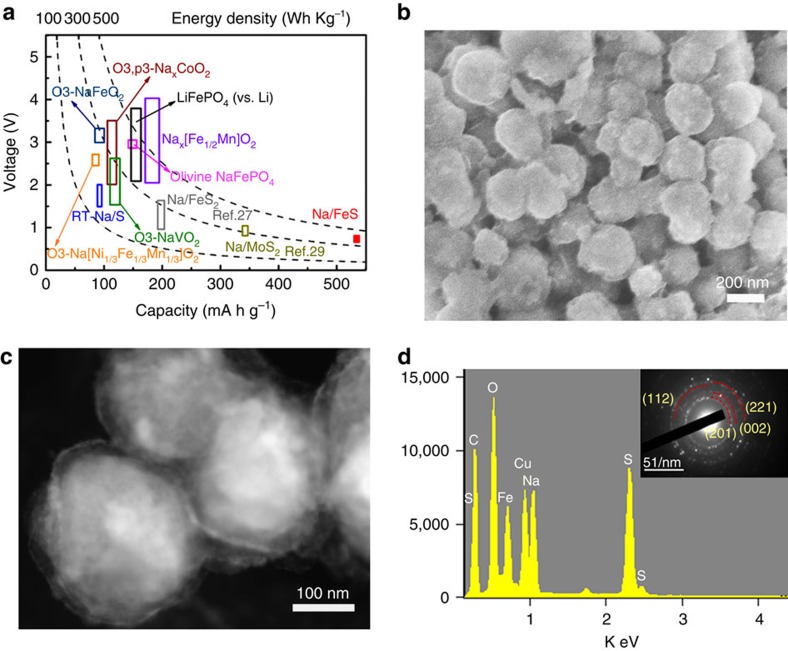
Superior energy density and morphological changes of the Na-FeS@C cells. (**a**) Average voltage and energy density versus gravimetric capacity of sodium-storage technologies (LiFePO_4_ plotted for reference). (**b**) SEM image, (**c**) scanning TEM image and (**d**) corresponding energy-dispersive spectroscopy spectrum and SAED pattern (inset) of the yolk-shell FeS@C electrode after 50 cycles at the current rate of 0.2 C.

The superiority of the Na/FeS battery is further highlighted by its energy density (Fig. 5a). The commercially available LiFePO_4_ cathode in LIBs is displayed as a reference standard, which can achieve a high energy density of ∼530 Wh kg^−1^. It is obvious that the energy densities of most NIB cathodes and of the RT-Na/S battery are <300 Wh kg^−1^. Surprisingly, by integrating the area underneath the discharge curve in Supplementary Fig. 13, the Na/FeS system in this work stands out among all those energy-storage technologies, as it retains a capacity of ∼545 mA h g^−1^ over 100 cycles, reaching an energy density as high as ∼438 Wh kg^−1^, exceeding those of other sodium-based batteries. Furthermore, the morphological and compositional changes in the uniform yolk-shell FeS@C nanosphere-based electrode after 50 cycles were investigated via SEM and TEM. Theoretically, the volume changes of FeS during sodiation/desodiation processes are estimated according to the following reaction:


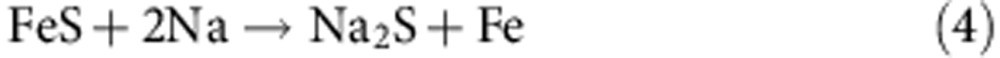


Assuming 1 mol of FeS reaction, the volume of FeS, Na_2_S and Fe can be calculated to be *V*_FeS_=*M*_FeS_/*ρ*_FeS_=18.16 cm^3^, *V*_Fe_=7.1 cm^3^ and *V*_Na2S_=41.96 cm^3^, respectively. The volume variation is calculated to be ∼170%; thus, the void space is required to be ∼16.5 nm to accommodate the volume expansion of the FeS yolk (∼170 nm). The designed extra void space (∼20 nm), therefore, is supposed to be necessary and sufficient to tolerate the volume changes in the FeS yolk. As revealed in Fig. 5b, the electrode retains the particle structure of fresh FeS@C nanospheres, although it is more like the core-shell structure, mostly because the pulverized and amorphous active materials would occupy the void space due to the conversion reaction. The scanning TEM image (Fig. 5c) shows an obvious core-shell structure; all the void spaces are occupied by the swollen FeS yolk. In agreement with our expectations, these results clearly indicate that the void spaces (∼20 nm and 1.88 times larger than the volume of the yolk FeS crystal) are large enough to accommodate the volume expansion of FeS particles during the charge/discharge process. As shown in the scanning TEM images (Fig. 5c,d), the energy-dispersive spectroscopy spectrum shows intense signals of the elements Fe, S, Na, C and O, although the corresponding SAED (inset) rings could only be indexed to the (201), (002), (112) and (211) plane of crystalline Na_2_CO_3_. This implies that the products of the Na/FeS cell via the conversion reaction are amorphous or ultra-fine and only the Na_2_CO_3_ formed during SEI formation is detectable. In comparison, serious electrode cracking is observed for both the micro-FeS electrode and the core-shell FeS/C electrode. The FeS particles in the micro-FeS electrode are wholly destroyed and the electrode shows significant structural cracking and pulverization (Supplementary Fig. 14). The core-shell structure is serious degenerated as well; fractures of the carbon shells and agglomerated FeS cores can be easily observed (Supplementary Fig. 15), resulting in the above inferior Na-storage properties. Owing to the unique yolk-shell structure, the free and accessible voids of the FeS@C nanocomposite spheres are large enough and can tolerate the large volume changes of the FeS core and maintain its original morphology. The carbon shells can improve the conductivity and help to form a stable SEI layer. The unique yolk-shell Na/FeS@C composite cell, therefore, is capable of achieving excellent cycling stability and rate capability.

Furthermore, based on this spatially confined sulfuration strategy, it can be demonstrated that the key factors for successfully constructing unique yolk-shell metal sulfide@C include high quality of the yolk-shell metal oxide@C precursor, the porosity of the carbon shells and sufficient void space between the yolk and shell to accommodate the resultant sulfur. On the other hand, the solid-state reaction temperature is vital for forming the target product. For instance, in this work, different morphologies of FeS_2_ would be formed from the solid-state reaction of the Fe_3_O_4_ nanospheres with the impregnated sulfur at 350 °C and 450 °C instead of FeS crystals at 550 °C (Supplementary Fig. 16). We hope that we have also presented a general method for the fabrication of yolk-shell metal sulfide@C nanocomposite via the spatially confined sulfuration strategy, which is expected to be widely extended to a broad class of metal sulfides such as SnS_*x*_, MoS_*x*_, WS_*x*_ and CoS_*x*_.

## Methods

### Synthesis of Fe_3_O_4_ nanospheres

The Fe_3_O_4_ nanospheres were synthesized through a solvothermal method using trisodium citrate as the stabilizer and FeCl_3_ as the iron source in ethylene glycol solution. Specifically, 3.25 g FeCl_3_ 6H_2_O, 1.3 g trisodium citrate and 1.0 ml H_2_O were dissolved in 100 ml ethylene glycol by stirring for 1 h; 6.0 g sodium acetate (NaAc) was then added into the solution. After magnetic stirring for 1 h and ultrasonication for 0.5 h, the obtained yellow solution was then sealed in a Teflon-lined autoclave. The autoclave was heated at 200 °C for 10 h and then naturally cooled down to room temperature. The Fe_3_O_4_ nanospheres were collected by a magnet and then washed three times with deionized water and ethanol.

### Synthesis of Fe_3_O_4_@SiO_2_ nanospheres

Fe_3_O_4_ (0.15 g) nanoparticles obtained above was well dispersed in 194 ml isopropyl alcohol after 0.5 h ultrasonication and 0.5 h stirring, followed by adding 18 ml H_2_O, 10 ml NH_4_OH and 0.2 ml tetraethyl orthosilicate to the flask. The dispersion was further stirred for 2 h at 40 °C. The core-shell Fe_3_O_4_@SiO_2_ nanospheres were collected by a magnet and then washed three times with deionized water and ethanol.

### Synthesis of Fe_3_O_4_@C nanospheres

An aqueous dispersion containing 0.15 g Fe_3_O_4_@SiO_2_ particles, 0.46 g cetyltrimethylammonium bromide and 14.08 ml H_2_O was transferred into a three-neck round-bottom flask. After 0.5 h ultrasonication and 1 h stirring, 0.7 g resorcinol, 56.4 ml absolute ethanol and 0.2 ml NH_4_OH were added sequentially. The flask was stirred for 0.5 h at 35 °C and then 0.1 ml formalin (F) was finally added. After continually stirring for 6 h and polymerization via ageing overnight, the obtained Fe_3_O_4_@SiO_2_@RF nanospheres were collected by a magnet and washed three times with deionized water and alcohol. The core-shell Fe_3_O_4_@SiO_2_@C was prepared by calcination of the Fe_3_O_4_@SiO_2_@RF powder at 600 °C for 3 h in N_2_ atmosphere. Finally, the silica layer was etched away by a 1.0 M NaOH solution, to yield the yolk-shell Fe_3_O_4_@C nanospheres.

### Synthesis of yolk-shell and core-shell FeS-C nanospheres and micro-FeS

A mixture of Fe_3_O_4_@C:sulfur in weight ratio of 1:1.1 was first ground by mortar and pestle, and then sealed in a Teflon-lined autoclave. The Fe_3_O_4_-S@C composite was obtained after the autoclave was heated at 155 °C for 12 h under air atmosphere. Then, the Fe_3_O_4_-S@C composite was sealed in a stainless steel tube. Finally, the yolk-shell FeS@C nanospheres (FeS@C) were fabricated via heat treatment in the stainless steel tube at 550 °C for 2 h in N_2_ atmosphere. For comparison, the micro-FeS and core-shell FeS/C nanospheres (FeS/C) were synthesized by the same procedures with core-shell Fe_3_O_4_/C or Fe_3_O_4_ nanoparticles with S as starting materials, respectively.

### Structural characterization

The morphologies of the samples were investigated by field-emission SEM (JEOL JSM-7500FA) and TEM (JEOL 2011, 200 keV). Raman spectra were collected by a 10-mW helium/neon laser at 632.8 nm excitation, which was filtered by a neutral density filter to reduce the laser intensity and a CCD (charge-coupled detector). The XRD patterns were collected by powder XRD (GBC MMA diffractometer) with Cu Kα radiation at a scan rate of 2 and 0.5 degree per minute. The XPS experiments were carried out using Al K*α* radiation and fixed analyser transmission mode. The pass energy was 60 eV for the survey spectra and 20 eV for the specific elements. The XPS samples were stored in an argon-filled glove box before testing to avoid oxidation.

### Electrochemical measurements

The electrochemical tests were conducted by assembling coin-type half-cells in an argon-filled glove box. The electrode slurry was prepared by fully mixing 80 wt% active materials (M-FeS, core-shell FeS/C or yolk-shell FeS@C), 10 wt% carbon black and 10 wt% carboxymethyl cellulose in an appropriate amount of water by planetary mixer (KK-250S). Then, the obtained slurry was pasted on copper foil using a doctor blade with a thickness of 100 μm, which was followed by drying at 80 °C in a vacuum oven overnight. The working electrode was prepared by punching the electrode film into discs 0.97 cm in diameter; the loading amounts of active materials on each electrode disc were ∼1.5–2.0 mg cm^−2^. The sodium foil was cut using a surgical blade from sodium bulk stored in mineral oil. The sodium foil was employed as both reference and counter electrode. The electrodes were separated by a glass fibre separator. Electrolyte consisting of 1.0 M NaClO_4_ in propylene carbonate/ethylene carbonate with a volume ratio of 1:1 and 5 wt% fluoroethylene carbonate additive (PC/EC+5 wt% FEC) was prepared and used in this work. The electrochemical performances were tested on a LAND Battery Tester within a voltage window of 0.01–2.3 V. All the capacities of cells have been normalized based on the weight of active materials in the form of FeS for the micro-FeS, and FeS-C nanocomposite for the core-shell FeS/C and the yolk-shell FeS@C. Cyclic voltammetry was performed using a Biologic VMP-3 electrochemical workstation.

## 

## Additional information

**How to cite this article:** Wang, Y.-X. *et al.* Uniform yolk-shell iron sulfide–carbon nanospheres for superior sodium–iron sulfide batteries. *Nat. Commun.* 6:8689 doi: 10.1038/ncomms9689 (2015).

## Supplementary Material

Supplementary Information Supplementary Figures 1-16
