# Supplementary material for: Uniform yolk-shell iron sulfide–carbon nanospheres for superior sodium–iron sulfide batteries
Source: Nat Commun. 2015 Oct 28;6:8689. doi: 10.1038/ncomms9689 (PMC4846313; doi:10.1038/ncomms9689)
Supplement: Supplementary Information — Supplementary Figures 1-16 [file ncomms9689-s1.pdf]

Supplementary Figures

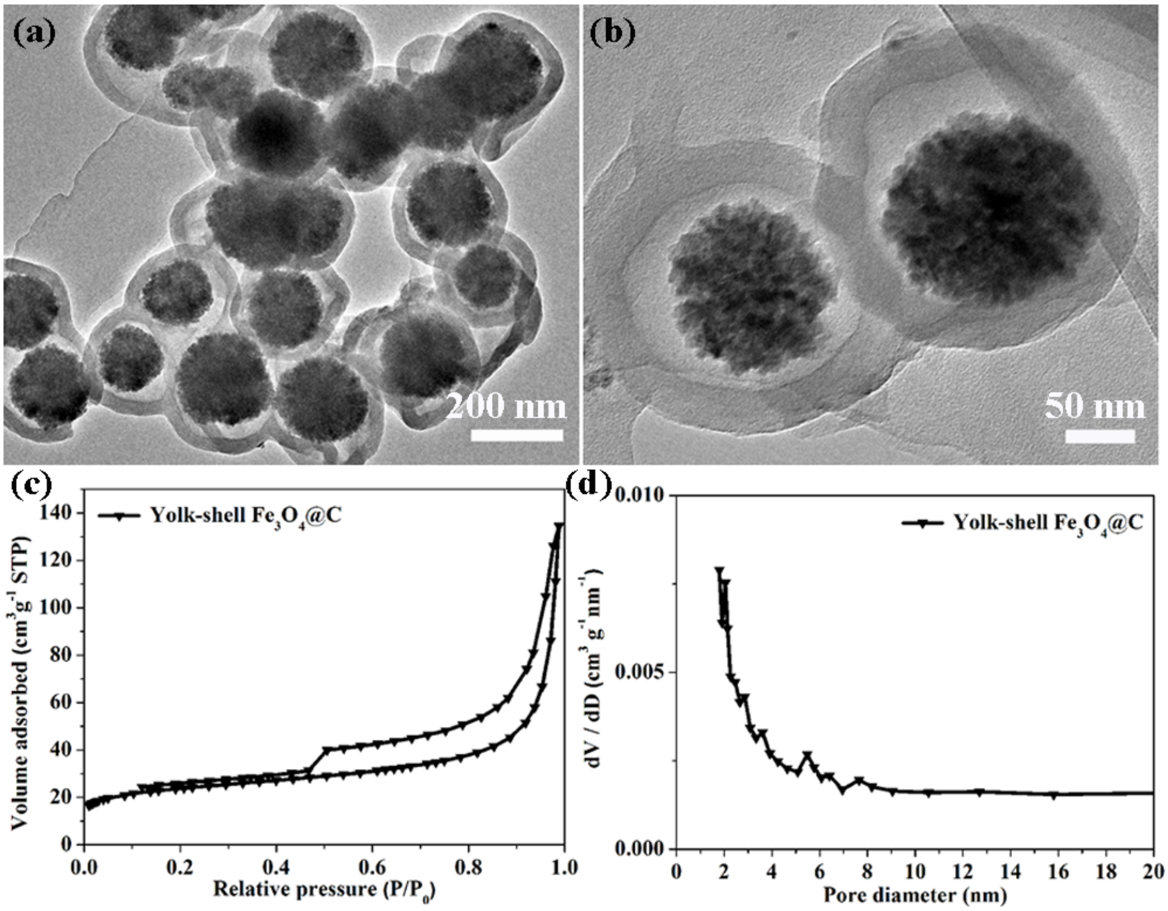

Supplementary Figure 1.TEM images (a) and (b) with different magnification of the yolk-shell structured Fe_3O_4@C nanospheres by etching away the condense SiO_2middle layer from the core-shell Fe_3O_4@SiO_2@C nanocomposites. (c) N_2 sorption isotherms and (d) pore size distribution curve of the yolk-shell Fe_3O_4@C nanospheres. The Brunauer-Emmett-Teller (BET) surface area, total pore volume, and pore size distribution of the yolk-shell Fe_3O_4@C nanospheres are calculated to be as high as 138 m^2g^{-1}, 0.21 cm^3g^{-1}, and 2 nm, respectively.

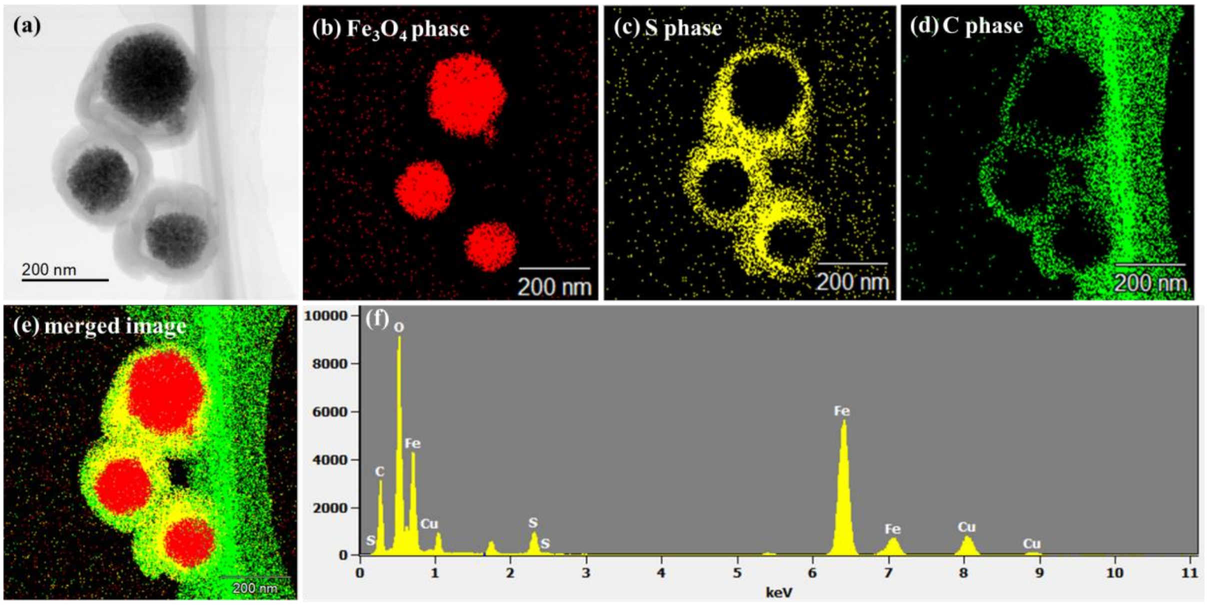

Supplementary Figure 2. (a) TEM image, the corresponding phases mapping of (b) Fe_3O_4, (c) S, (d) C, and the merged image, and the corresponding EDS spectrum for the core-shell Fe_3O_4-S@C nanocomposites after loading sulfur into yolk-shell Fe_3O_4@C *via* melt-diffusion strategy. The phase mapping images demonstrate that the sulfur is well-loaded in the void of yolk-shell Fe_3O_4@C nanospheres.

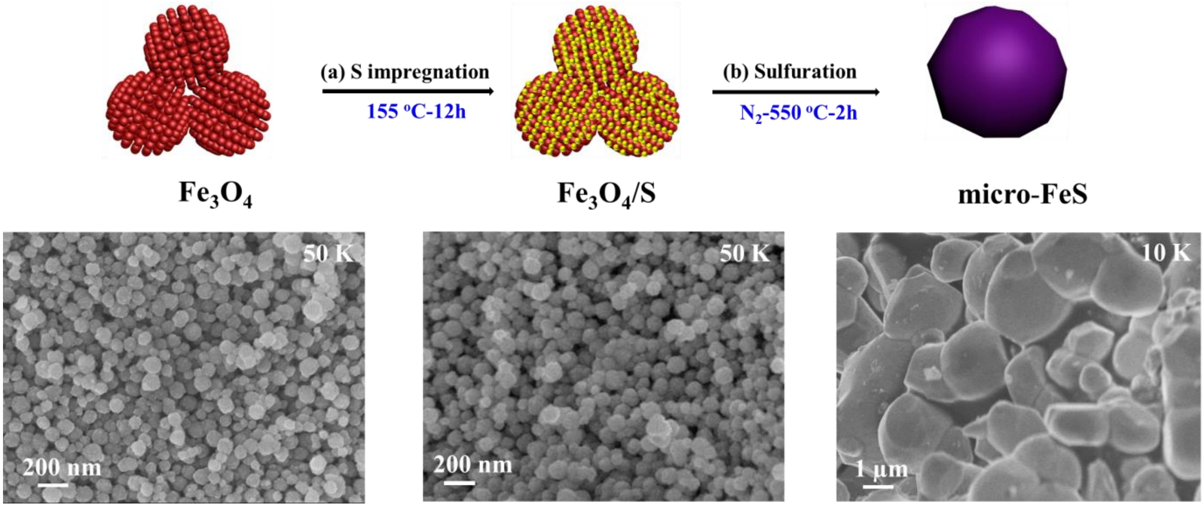

Supplementary Figure 3. Schematic illustration for the preparation process of the micro-sized FeS particles (micro-FeS) and the corresponding SEM images of the Fe_3O_4 nanoparticles, the Fe_3O_4/S mixture, and micro-FeS.

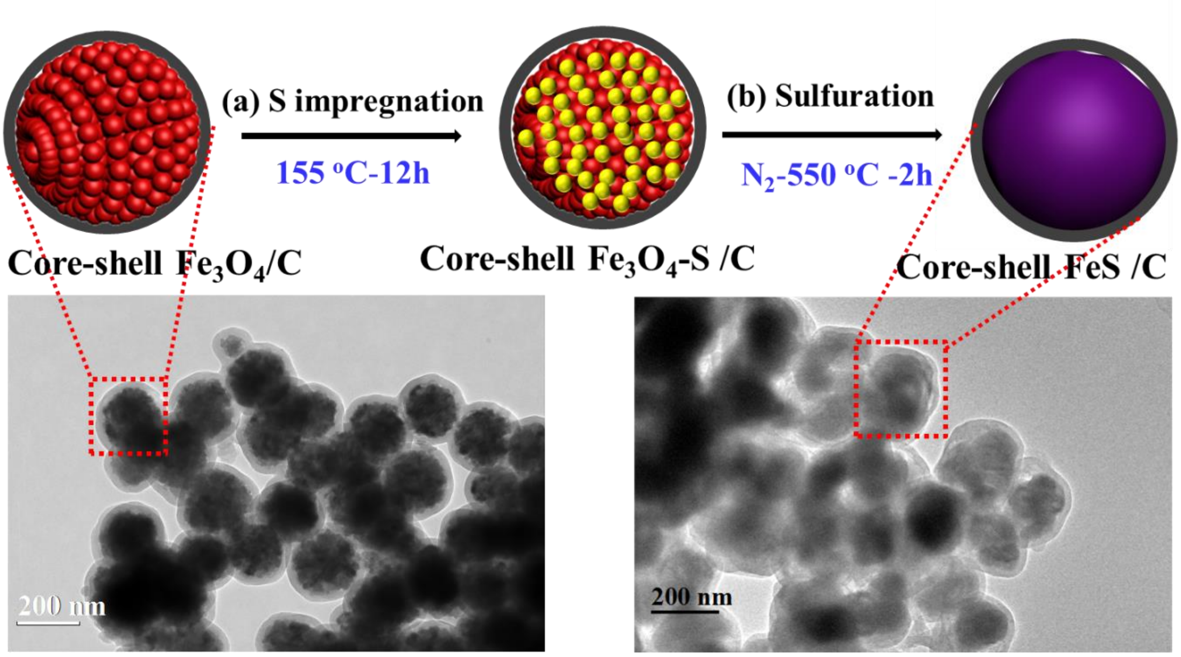

Supplementary Figure 4. Schematic illustration for the preparation process of the core-shell FeS/C nanoparticles (FeS/C) and the corresponding TEM images of the core-shell Fe_3O_4/C and FeS/C nanoparticles.

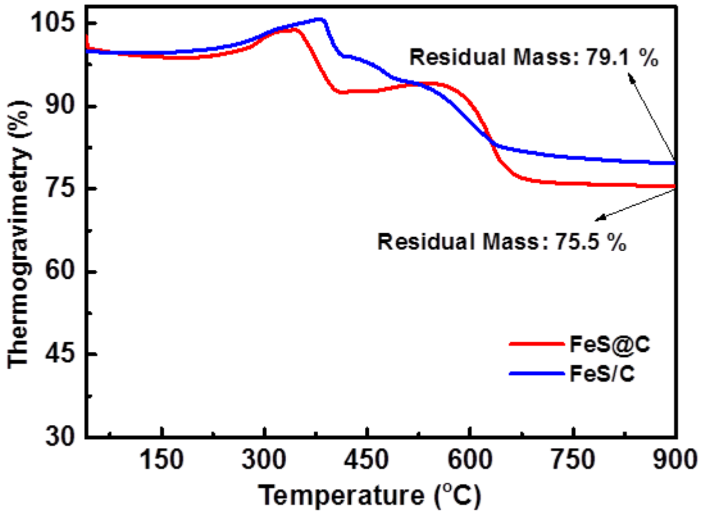

Supplementary Figure 5. TGA results of yolk-shell FeS@C and core-shell FeS/C nanocomposites.

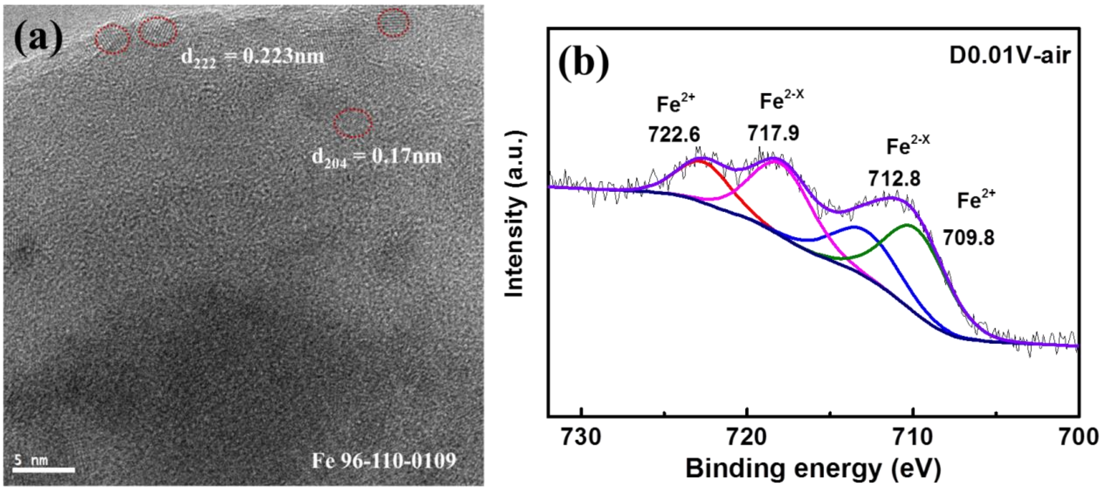

Supplementary Figure 6. The corresponding TEM image of micro-FeS electrodes at discharged to 0.01 V (D0.01 V) and the ex-situ XPS spectra of Fe 2p for the D0.01 V electrode exposed to air overnight (D0.01 V-air).

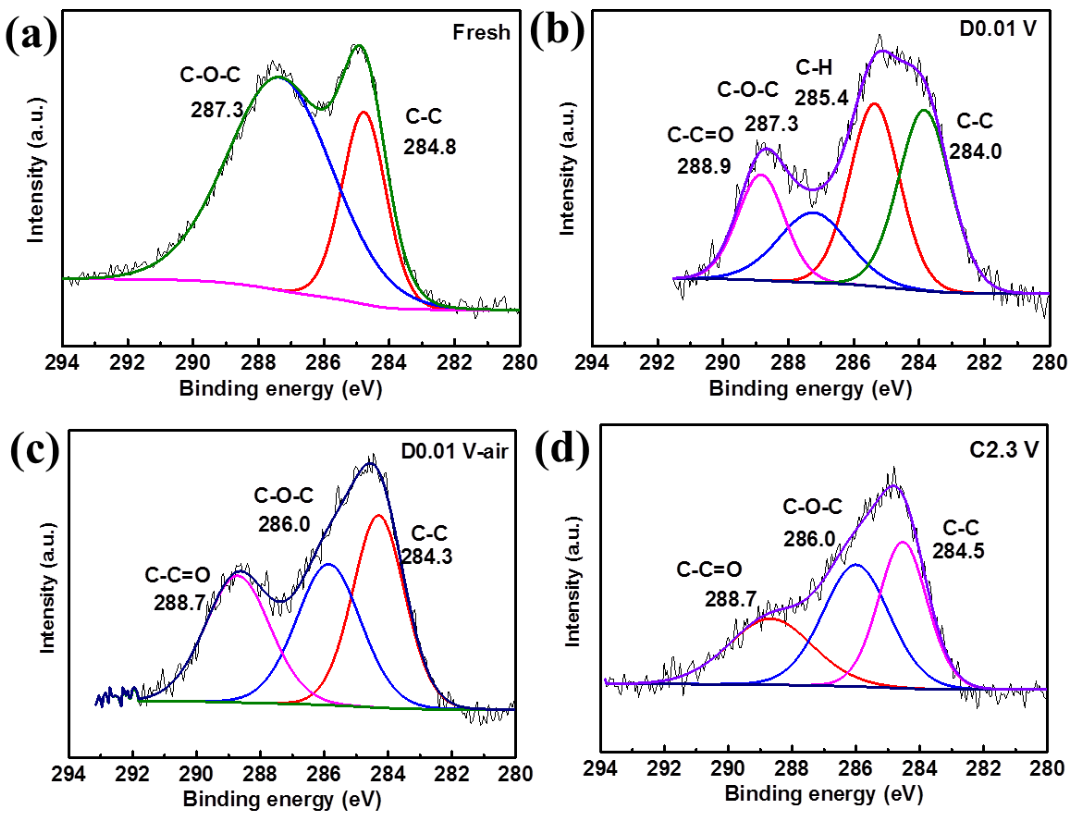

Supplementary Figure 7. The respective *ex-situ* XPS spectra of C1s from the micro-FeS electrodes at (a) fresh state, (b) discharged to 0.01 V (D0.01 V), (c) the D0.01 V electrode exposed to air overnight (D0.01 V-air), and (d) charged back to 2.3V (C2.3 V).

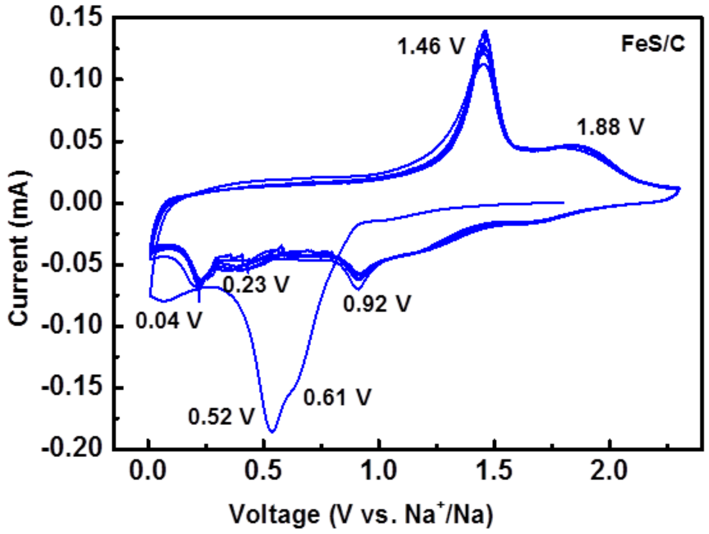

Supplementary Figure 8. Cyclic voltammograms of the core-shell FeS/C nanospheres.

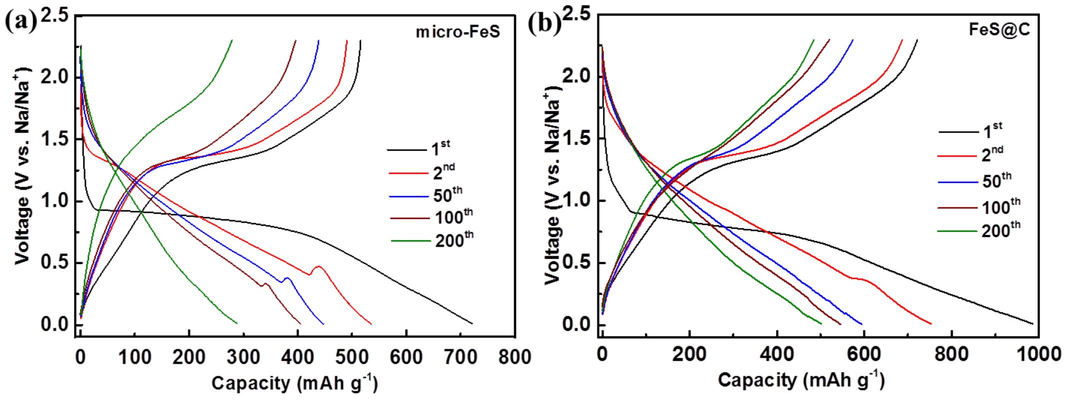

Supplementary Figure 9. Charge/discharge curves of (a) the micro-FeS and (b) yolk-shell FeS@C for the selected cycles at 100 mAh g^{-1}.

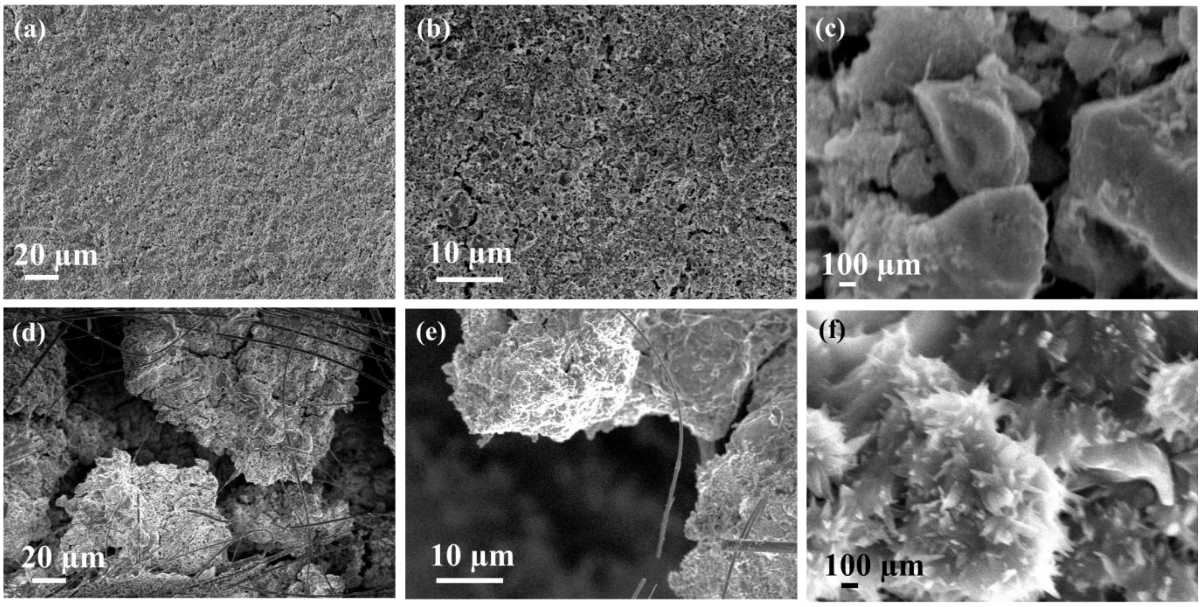

Supplementary Figure 10. SEM images of the micro-FeS-based electrodes (a, b, and c) before and (d, e, and f) after 150 cycles at different magnifications.

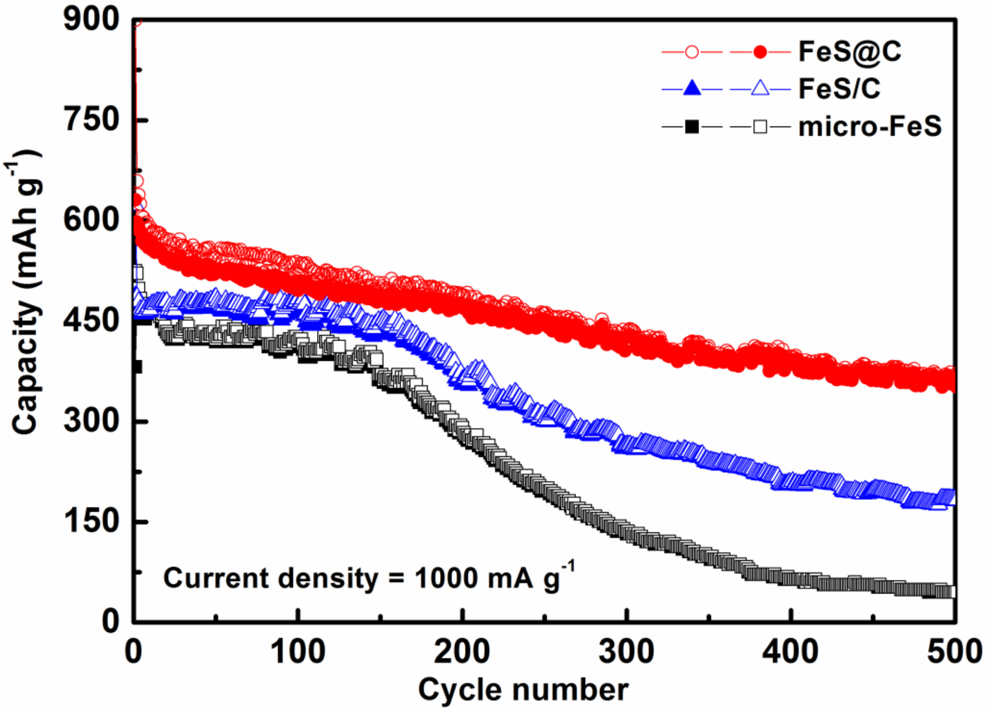

Supplementary Figure 11. High-rate cycling performance of the micro-FeS, core-shell FeS/C nanospheres, and yolk-shell FeS@C nanospheres over 500 cycles at the current rate of 2 C (1000 mA g^{-1}).

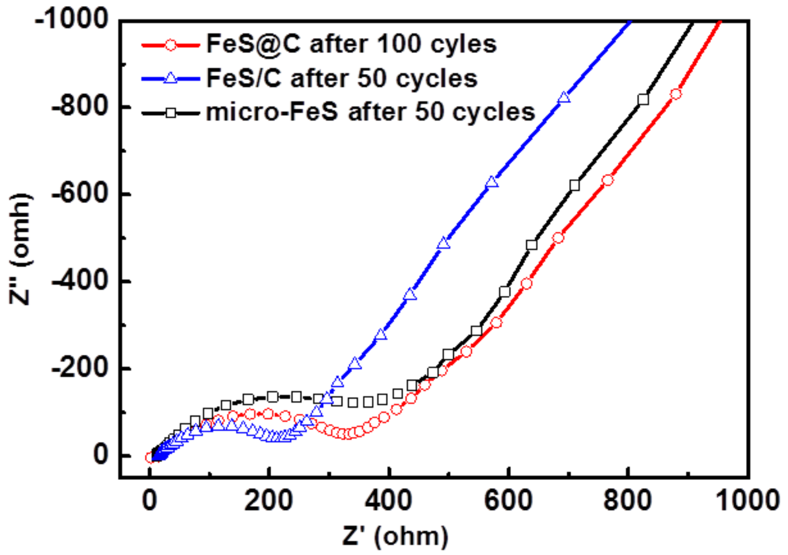

Supplementary Figure 12. The impedance plots of the micro-FeS particles, the core-shell FeS/C nanospheres, and the yolk-shell FeS@C nanospheres after rate capability testing at various current densities.

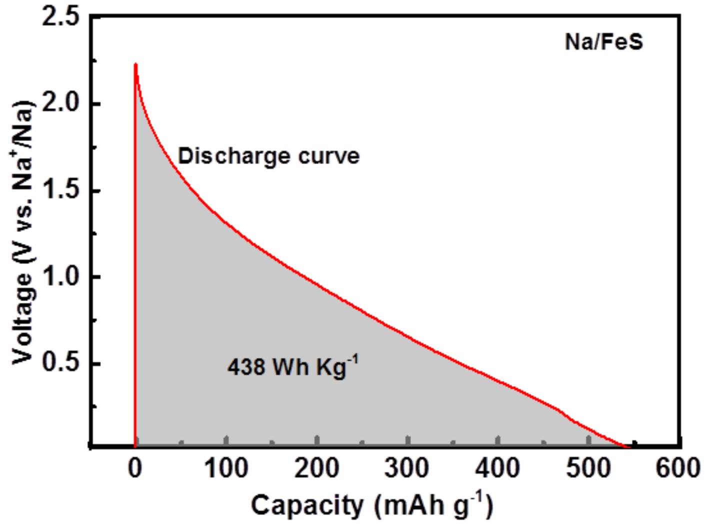

Supplementary Figure 13. The calculation of energy density for Na/FeS@C after 100 cycles.

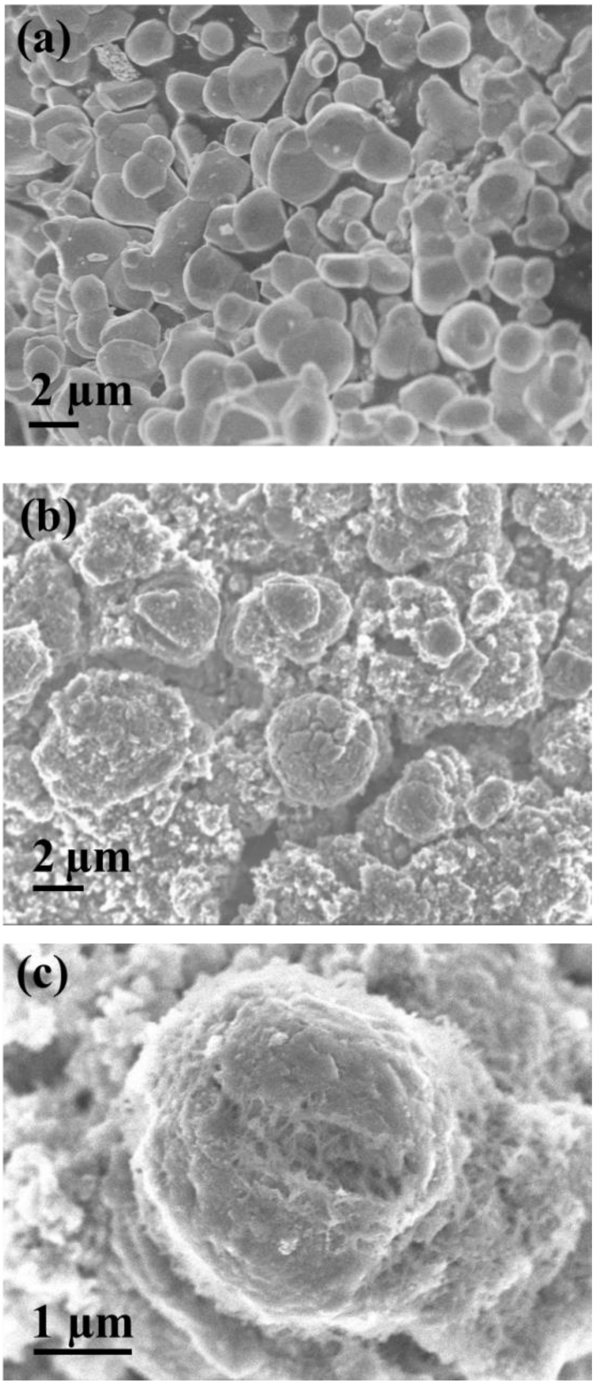

Supplementary Figure 14. SEM images of the micro-FeS-based electrodes (a) before and (b), (c) after 50 cycles at different magnifications.

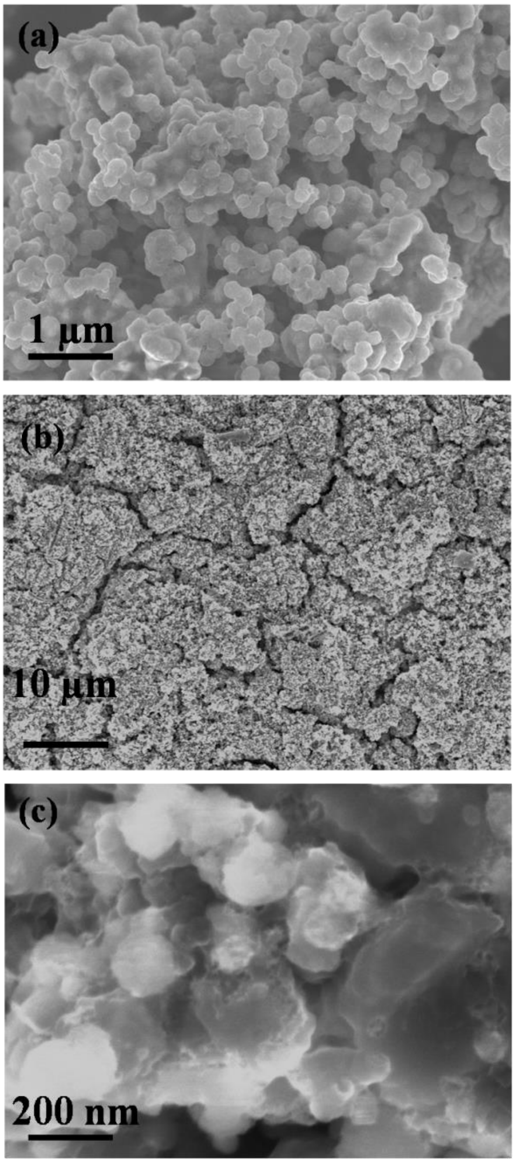

Supplementary Figure 15. SEM images of the core-shell FeS/C-based electrodes (a) before and (b), (c) after 50 cycles at different magnifications.

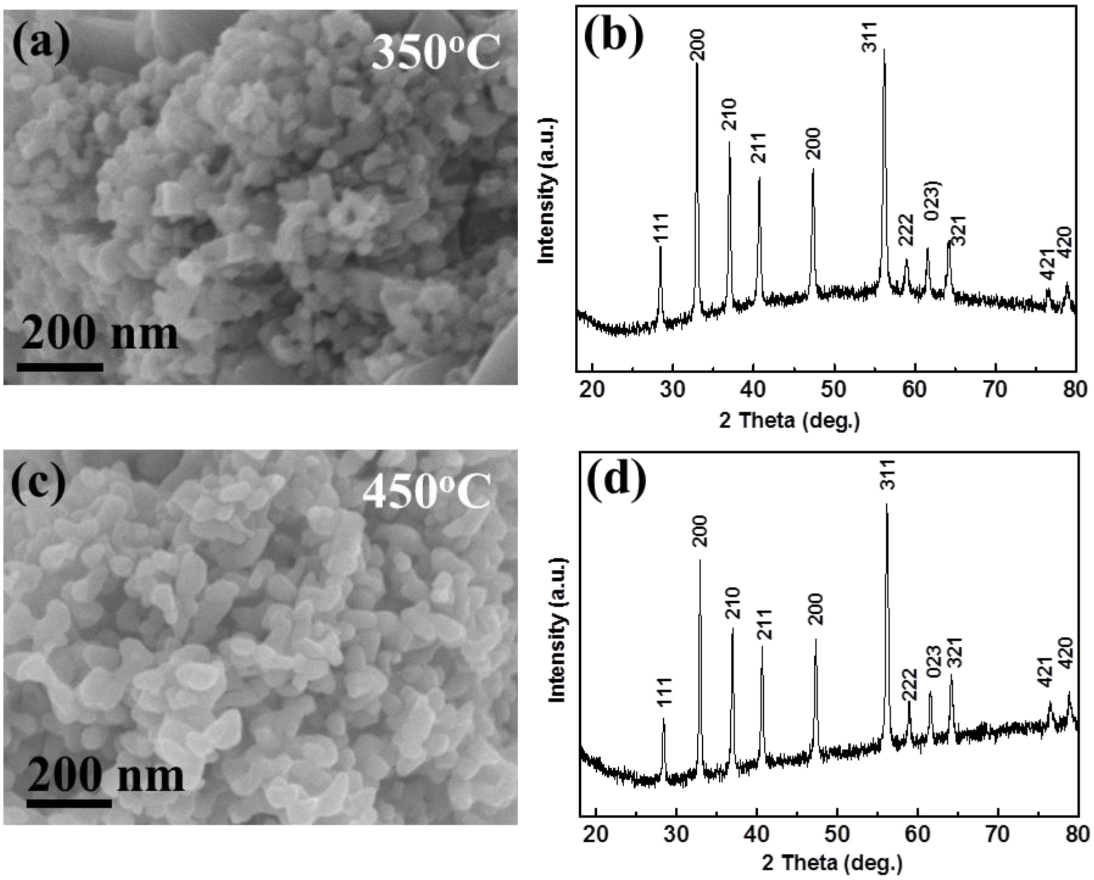

Supplementary Figure 16. SEM images (a) and (c); XRD patterns (b) and (d) of FeS_2 nanoparticles prepared by sulfuration reaction of the Fe_3O_4/S mixture at 350 and 450°C, respectively.
